# CRB2 Facilitates Epithelial Ovarian Cancer Progression by Inducing Polarity Changes via Activation of the Wnt/PCP Signalling Pathway

**DOI:** 10.1111/jcmm.71124

**Published:** 2026-03-29

**Authors:** Chunlin Tao, Shaojing Li, Duohe Sun, Jun Zhou, Yuanping Chen, Rong Zhang, Xiaoge Ni

**Affiliations:** ^1^ Department of Obstetrics and Gynaecology Shanghai Jiao Tong University Affiliated Sixth People's Hospital South Campu Shanghai China; ^2^ Graduate Student Training Base of Jinzhou Medical University (Shanghai Fengxian District Central Hospital) Shanghai China; ^3^ Department of Obstetrics Obstetrics & Gynaecology Hospital of Fudan University, Yangtze River Delta Integration Demonstration Zone(QingPu) Shanghai China; ^4^ Department of Gynecologic Oncology Shanghai Geriatric Medical Center Shanghai China; ^5^ Department of Gynaecology, Shanghai Xuhui Central Hospital Zhongshan‐ Xuhui Hospital, Fudan University Shanghai China

**Keywords:** cell polarity, crumbs cell polarity complex component 2 (CRB2), epithelial ovarian cancer (EOC), Wnt/planar cell polarity (Wnt/PCP) pathway

## Abstract

Ovarian cancer exhibits high molecular heterogeneity and metastatic potential, contributing to its status as a leading cause of gynecologic cancer mortality. Cell polarity is essential in tumorigenesis, yet the role of Crumbs family proteins (CRBs), key regulators of apical–basal polarity, in epithelial ovarian cancer (EOC) remains unclear. In this study, we analysed CRB expression profiles using TCGA and GEO datasets, and validated our findings through immunohistochemical staining of ovarian tumour tissue microarrays. Among CRBs, CRB2 was significantly overexpressed in EOC tissues and correlated with poor patient prognosis. Functional assays revealed that CRB2 enhances ovarian cancer cell proliferation, migration, and invasion, while suppressing apoptosis. Immunofluorescence staining of planar cell polarity markers demonstrated that CRB2 induces polarity alterations in EOC cells. Furthermore, Western blot analysis suggested that CRB2 may activate the Wnt/planar cell polarity (PCP) signalling pathway, contributing to tumour progression. These findings identify CRB2 as a key modulator of cell polarity and a potential driver of EOC aggressiveness. CRB2 may serve as a novel prognostic biomarker and therapeutic target for epithelial ovarian cancer.

## 1 Introduction

Ovarian cancer accounts for approximately 3.4% of all female cancers and 4.8% of cancer‐related deaths among women, ranking eighth in global female cancer mortality and second among gynecologic malignancies [1]. In the United States, an estimated 20,890 new cases of ovarian cancer and 12,730 related deaths are expected in 2025 [2]. In China, there were approximately 61,100 new cases and 32,600 deaths due to ovarian cancer in 2022 [3], with the average annual percentage change (AAPC) of incidence and mortality continuing to show a slow upward trend [4]. Epithelial ovarian cancer (EOC) is the most common subtype, accounting for about 90% of all ovarian cancer cases. Metastasis remains the primary cause of cancer‐related death [5, 6]. During the metastatic process, solid tumour cells acquire various types of polarity, such as apical–basal polarity and front–rear polarity [7, 8].

The Crumbs family in vertebrates consists of three members: CRB1, CRB2, and CRB3. Crumbs proteins are key regulators of epithelial development and tissue homeostasis [9]. They form the core of the Crumbs complex by interacting with PALS1 and either PATJ or MUPP1 [10]. Together with two other evolutionarily conserved polarity protein complexes—Scribble and PAR—the Crumbs complex constitutes essential apical membrane landmarks. These complexes orchestrate proper cytoskeletal organisation and regulate apical–basal polarity, ensuring precise control of cell polarity [11, 12, 13, 14, 15].

Previous studies have reported that mutations in the CRB1 gene can lead to various forms of systemic retinal dystrophies [16, 17, 18, 19]. CRB2 is significantly upregulated in human glioblastoma (GBM) and has been associated with poor prognosis [20]. CRB3 expression is correlated with estrogen and progesterone receptor positivity in breast cancer [21], promotes cell migration in colorectal cancer [22, 23], and serves as a prognostic biomarker in clear cell renal cell carcinoma [24].

However, the functional role and clinical significance of Crumbs family proteins (CRBs) in epithelial ovarian cancer (EOC) remain largely uncharacterised. Considering their established involvement in tumour progression and cell polarity regulation in other cancer types, further investigation is warranted to elucidate their potential contributions to EOC pathogenesis.

## 2 Materials and Methods

### 2.1 Data Collection and Processing

In this study, differential gene expression analysis between ovarian tumour tissues and normal ovarian tissues was conducted using data from The Cancer Genome Atlas (TCGA) combined with the Genotype‐Tissue Expression (GTEx) database to identify candidate genes. Gene expression levels of CRB1, CRB2, and CRB3 in TCGA ovarian cancer (OV) and GTEx normal ovarian tissues were analysed using GEPIA2 (http://gepia2.cancer‐pku.cn/). Data are shown as log2(TPM + 1); P values were calculated by two‐tailed Student's *t*‐test (*p* < 0.05). Subsequently, the expression profiles of the candidate gene across pan‐cancer datasets were analysed using R software to confirm its statistically significant overexpression in ovarian cancer. Further in‐depth analysis of the candidate gene's expression in normal ovarian and ovarian cancer tissues was performed using multiple datasets from the Gene Expression Omnibus (GEO) database. Finally, survival analysis was carried out using the Kaplan–Meier Plotter database to evaluate the impact of candidate gene overexpression on progression‐free survival (PFS) and overall survival (OS) in ovarian cancer patients.

### 2.2 Patient Specimens

A total of 328 ovarian tissue samples were collected for this study, including 154 benign ovarian epithelial cyst cases and 174 epithelial ovarian cancer (EOC) cases from female patients aged 15 to 82 years. All specimens were obtained between 2012 and 2020 at the Shanghai Jiao Tong University Affiliated Sixth People's Hospital South Campus. None of the patients received any treatment prior to surgery. The collection of all samples and the study design were approved by the Ethics Committee of Shanghai Jiao Tong University Affiliated Sixth People's Hospital South Campus, in accordance with the ethical principles outlined in the Declaration of Helsinki.

### 2.3 Immunohistochemistry (IHC) Analysis

Paraffin‐embedded tissue sections or tissue microarrays were deparaffinised, rehydrated, and subjected to antigen retrieval. Endogenous peroxidase activity was quenched, followed by blocking with bovine serum albumin (BSA) at room temperature for 1 h. Sections were then incubated overnight at 4°C with the primary antibody. On the following day, slides were incubated with goat anti‐rabbit IgG secondary antibody at room temperature for 1 h. Colour development was performed using a horseradish peroxidase (HRP) substrate, followed by haematoxylin counterstaining. Differentiation was carried out with 1% HCl in 75% ethanol, followed by continuous rinsing under running water to achieve bluing. Finally, the sections were dehydrated, dried, and mounted. The staining intensity and the percentage of positive tumour cells were scored semi‐quantitatively. Staining intensity was graded as 0 (negative), 1 (weak), 2 (moderate), and 3 (strong). The proportion of positive cells was scored as 0 (0%–5%), 1 (6%–25%), 2 (26%–50%), 3 (51%–75%), and 4 (> 75%). The final IHC score was calculated as the product of the intensity and proportion scores (range: 0–12). Based on the total score, samples were categorised into low (≤ 6) and high (> 6) CRB2 expression groups. All slides were independently evaluated by two experienced pathologists in a blinded manner, and discrepancies were resolved by joint review.

### 2.4 Cell Culture

Human ovarian cancer cell lines used in this study were kindly provided by Professor Zhigang Zhang from the Cancer Institute of Shanghai Jiao Tong University. Cells were cultured in either RPMI‐1640 or DMEM medium (Solarbio, Shanghai), supplemented with 10% fetal bovine serum (FBS, Gibco, USA) and 100 μg/mL penicillin–streptomycin (Solarbio, Shanghai). All cell lines were maintained in a humidified incubator at 37°C with 5% CO₂. The ovarian cancer cell lines selected for this study included OVCAR8, ES2, SKOV3, and CAOV3.

### 2.5 Reagents and Antibodies

The reagents used in this study are detailed within the corresponding experimental procedures. The antibodies used include: CRB2 (ab175546, Abcam, USA), Wnt5a/b (55184–1‐AP), VANGL2 (21492–1‐AP), JUN (24909–1‐AP), JNK (24164–1‐AP), RAC1 (24072–1‐AP), β‐TUBULIN (80713–1‐RR), ATP1B1 (85916–1‐RR), and GM130 (11308–1‐AP), all purchased from Proteintech (Wuhan, China), FZD6 (DF4930) and DVL2 (DF4454), purchased from Affinity Biosciences (USA), and Cy3 Goat Anti‐Rabbit IgG (H + L) Antibody (K1209), obtained from APExBIO (USA).

### 2.6 Small Interfering RNA (siRNA) Interference Assay

Small interfering RNAs (siRNAs) were purchased from RiboBio (Shanghai, China). The sequences were as follows:

si‐NC (negative control):

forward: 5′‐UUCUCCGAACGUGUCACGUdTdT‐3′.

reverse: 5′‐GCGACGAUCUGCCUAAGAUdTdT‐3′.

si‐CRB2‐1:

forward: 5′‐CCGGCGCAGCTGGCCTAACAGTATTCTCGAGAA‐3′.

reverse: 5′‐TACTGTTAGGCCAGCTGCGTTTTTG‐3′.

si‐CRB2‐2:

forward: 5′‐CCGGTGTCTGAATG‐3′.

reverse: 5′‐TATCTGCAATGCTCGAGCATTGCAGATACATTCAGACATTTTTG‐3′.

Cell transfection was performed using Lipofectamine 3000 (Invitrogen, Carlsbad, CA, USA) according to the manufacturer's instructions.

### 2.7 Lentivirus Infection and Cell Transfection

CRB2 overexpression lentiviral plasmids, interference plasmids, and empty control vectors were obtained from OBiO Technology (China). Plasmids were transformed into *E. coli* DH5α competent cells (NCM Biotech, China) and plated on LB agar containing ampicillin. Single colonies were selected and expanded, and plasmids were extracted using an endotoxin‐free plasmid purification kit (DP118, TIANGEN, China). For lentiviral packaging, 4 μg of target plasmid, 2 μg of psPAX2 (Asia‐Vector, China), and 1 μg of pMD2.G (Asia‐Vector, China) were co‐transfected into HEK‐293 T cells using Lipofectamine 3000 and P3000 (Invitrogen, USA) in Opti‐MEM medium (Gibco, USA). The viral supernatant was harvested and used to infect ovarian cancer cell lines. Stable cell lines were selected using puromycin (Sigma, USA).

### 2.8 Quantitative Real‐Time PCR (qRT‐PCR) Analysis

Total RNA was extracted from cells using the Rapid RNA Extraction Kit (SB‐R001, Sharebio, China), and RNA concentration was measured using a NanoDrop spectrophotometer. For reverse transcription, 500 ng of total RNA was mixed with 2 μL of PrimeScript RT Master Mix (5×) (RR036A, Takara, Japan) and RNase‐free water to a total volume of 10 μL. The resulting cDNA was diluted 1:30 with RNase‐free water. For quantitative PCR, a 10 μL reaction mixture was prepared for each well in a 96‐well plate, containing 4.2 μL of diluted cDNA, 0.4 μL each of forward and reverse primers, and 5 μL of 2× SYBR Green qPCR Master Mix (B21402, Bimake, USA). Each sample was run in triplicate. The primers used in this study were as follows:

CRB2 forward: 5′‐ACCACTGTGCTTGTCCTGAG‐3′.

CRB2 reverse: 5′‐TCCAGGGTCGCTAGATGGAG‐3′.

RPS18 forward: 5′‐ATCACCATTATGCAGAATCCACG‐3′.

RPS18 reverse: 5′‐GACCTGGCTGTATTTTCCATCC‐3′.

### 2.9 Western Blot

Total protein was extracted from cells using RIPA lysis buffer (P0013B, Beyotime, China) supplemented with protease and phosphatase inhibitors (B15001, Bimake, USA). Membrane proteins were isolated using a membrane protein extraction kit (P0033, Beyotime, China). Protein concentrations were determined using a BCA protein assay kit (PC0020, ShareBio, China). Equal amounts of protein were separated by 10% SDS‐PAGE (PG112, EpiZyme, China) and transferred onto nitrocellulose (NC) membranes (Millipore, USA). Membranes were blocked with non‐fat milk at room temperature for 1 h, then incubated with primary antibodies overnight at 4°C. On the following day, membranes were incubated with HRP‐conjugated secondary antibodies at room temperature for 1 h. Protein bands were visualised using a general ECL detection reagent (LumiQ General, SB‐WB012, ShareBio, China) and imaged with a chemiluminescence detection system. Band intensities were quantified using ImageJ software.

### 2.10 Cell Counting Kit‐8 (CCK‐8) Assay

Cells in good condition and at the logarithmic growth phase were seeded into 96‐well plates at a density of 2000 cells per well, with six replicates for each condition. After incubation at 37°C for 24 h, the medium in each well was replaced with fresh medium containing CCK‐8 reagent (CX001S, Epizyme, China) at a ratio of 10 μL CCK‐8 per 100 μL medium. The plates were then incubated at 37°C in the dark for 1–4 h. Absorbance at 450 nm was measured using a microplate reader (Tecan, Switzerland) after equilibrating the machine to 37°C. Optical density (OD) values were recorded every 24 h for a total of 4–5 consecutive days.

### 2.11 Colony Formation Assay

Cells in good condition and at the logarithmic growth phase were seeded into 6‐well plates at a density of 1000 cells per well. After gently dispersing the cells into a single‐cell suspension, the plates were incubated for 7–14 days, with medium replaced every 2–3 days. When visible cell colonies formed from single ovarian cancer cells and became macroscopically detectable, the culture was terminated. Colonies were fixed with 4% paraformaldehyde for at least 30 min, then stained with 0.1% crystal violet for at least 30 min. After rinsing with running water to remove excess stain, plates were air‐dried and imaged under a microscope. Colony numbers were quantified using ImageJ software for statistical analysis.

### 2.12 Mouse Xenografts

All animal experiments were approved by the Ethics Committee of Shanghai Jiao Tong University Affiliated Sixth People's Hospital South Campus. Female BALB/c nude mice (6 weeks old, weighing 18–22 g) were purchased from Shanghai SLAC Laboratory Animal Co. Ltd. All mice were housed under specific pathogen‐free (SPF) conditions and cared for by trained personnel. All procedures followed the Helsinki Declaration and adhered to the Guidelines for the Care and Use of Laboratory Animals in China.

ES2 cells (with or without transfection) were subcutaneously injected into the right flank of nude mice (6 mice per group; 0.1 mL containing 5 × 10^6^ cells per mouse). Body weights were recorded every 2–3 days. After tumour formation, tumour length and width were measured regularly. Fifteen days post‐injection, the mice were euthanised, and subcutaneous tumours were excised for photography and further analysis. Tumour volume was calculated using the formula: Tumour volume (cm^3^) = (a^2^ × b)/2, where ‘a’ is the short diameter and ‘b’ is the long diameter. Tumour tissues were preserved for subsequent immunohistochemical analysis.

### 2.13 Transwell Migration Assay

Transwell chambers (Corning, USA) were placed in a 24‐well plate. Log‐phase cells in good condition were counted and resuspended in serum‐free medium. A total of 2 × 10^4^ cells in 200 μL of serum‐free medium were added to the upper chamber, while 700 μL of medium containing 10% fetal bovine serum (FBS) was added to the lower chamber as a chemoattractant. The chambers were incubated at 37°C in a 5% CO₂ incubator for 20–24 h. After incubation, cells on both sides of the membrane were fixed with 4% paraformaldehyde. The cells remaining on the upper side of the membrane were gently removed with a cotton swab. The migrated cells on the lower side were stained with 0.1% crystal violet, imaged under a microscope, and quantified. Six random fields were captured for each well, and the number of migrated cells was counted using ImageJ software for statistical analysis.

### 2.14 Transwell Invasion Assay

Matrigel matrix (354,234, Corning, USA) was thawed in advance at 4°C. An appropriate amount of Matrigel was diluted with pre‐cooled serum‐free DMEM at a ratio of 1:40. Then, 100 μL of the diluted Matrigel was added to the upper chamber of each Transwell insert. The inserts were incubated at 37°C for 2 h to allow the Matrigel to solidify and form a gel‐like membrane. After the Matrigel coating was completed, the remaining steps were performed in the same manner as described in the Transwell migration assay.

### 2.15 Flow Cytometry (Apoptosis Assay)

Cells in good condition and at logarithmic growth phase were seeded in 6‐well plates and incubated overnight. On the following day, the culture medium was replaced with fresh serum‐free medium to induce apoptosis by serum starvation for 24 h. On the third day, the medium from each well was collected, and the cells were digested with EDTA‐free trypsin. The collected medium was used to neutralise the trypsinisation. Cells were centrifuged to remove the supernatant, and apoptosis was assessed using an Annexin V‐FITC/PI apoptosis detection kit (C1052, Beyotime, China) according to the manufacturer's instructions. Flow cytometry analysis was performed using a BD flow cytometer (BD, USA).

### 2.16 Planar Polarity Immunofluorescence Staining

Cells in good condition and at logarithmic growth phase were seeded into 24‐well plates and incubated overnight. When cell confluence reached approximately 90% the next day, a vertical scratch was made using a 200 μL pipette tip with the aid of a ruler. Cells were washed twice with PBS to remove debris from the scratch, and fresh complete medium was added for continued incubation overnight. On the third day, immunofluorescence staining was performed. After removing the medium, cells were washed three times with PBS and fixed with 4% paraformaldehyde for at least 30 min. Cells were permeabilised with 0.1% Triton X‐100 (P0096, Beyotime, China) for 10 min, followed by incubation with a primary antibody (GM130, 1:50) diluted in 1% BSA at 4°C overnight. The next day, cells were incubated with a secondary antibody diluted in 1% BSA at room temperature in the dark for 1 h. Nuclei were counterstained with DAPI (1 μg/mL; C3362, APExBIO, USA) for 3–5 min. Finally, 1–2 drops of anti‐fade mounting medium were added per well, and cells were observed and imaged using an inverted fluorescence microscope.

### 2.17 Statistical Analysis

Statistical analyses were performed using IBM SPSS Statistics 25 and GraphPad Prism version 9 (San Diego, CA, USA). Data are presented as the mean ± standard deviation (SD) from at least three independent experiments. Comparisons between two groups were conducted using a two‐tailed Student's *t*‐test, while comparisons among multiple groups were performed using one‐way ANOVA. A *p*‐value less than 0.05 was considered statistically significant.

## 3 Results

### 3.1 Expression Profile and Survival Analysis of the Crumbs Family in Ovarian Cancer

**FIGURE 1:**
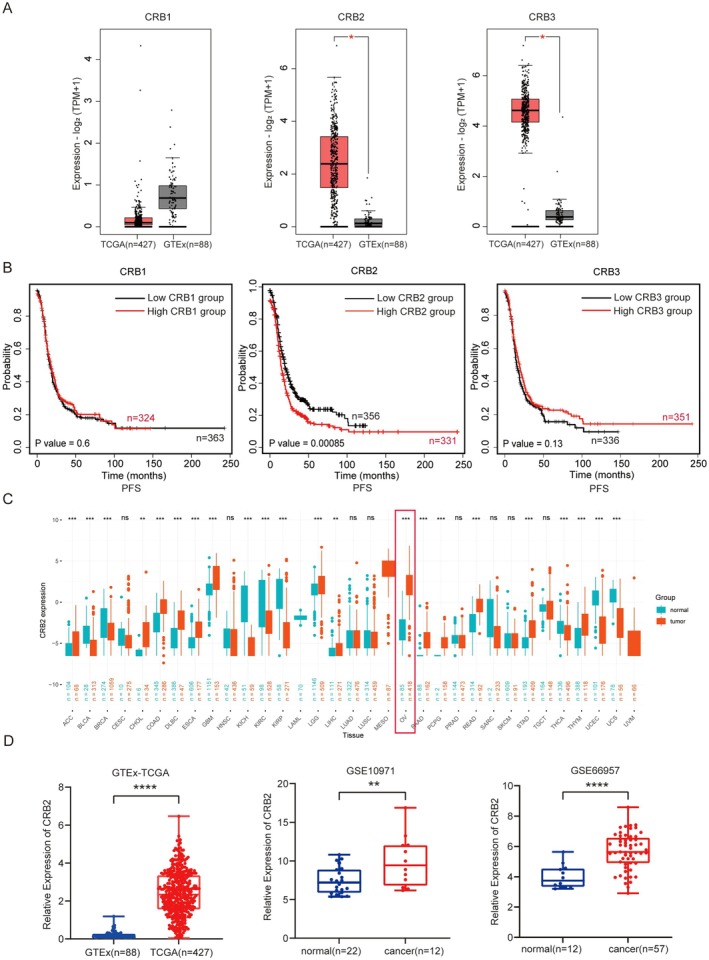
Expression Profile and Prognostic Significance of the Crumbs Family in Ovarian Cancer. (A) Expression levels of Crumbs family members in ovarian cancer tissues (TCGA) vs. normal ovarian tissues (GTEx). (B) Progression‐free survival analysis of Crumbs family members in ovarian cancer patients using the Kaplan–Meier Plotter database. (C) Pan‐cancer expression analysis of CRB2. (D) Validation of CRB2 expression across multiple datasets, including TCGA/GTEx and GEO datasets (GSE10971, GSE66957). (*p < 0.05, **p < 0.01, ***p < 0.001, ****p < 0.0001).

Expression levels of the Crumbs family were analysed in 88 normal ovarian tissues from the GTEx database and 427 ovarian cancer tissues from the TCGA database. CRB2 and CRB3 were found to be significantly upregulated in ovarian cancer tissues compared to normal ovarian tissues (*p* < 0.0001, Figure 1A). Using the Kaplan–Meier Plotter database, survival analysis revealed that high CRB2 expression was associated with poorer progression‐free survival (PFS) in ovarian cancer patients (*p* = 0.00085), whereas no significant differences in PFS were observed between high and low expression groups of CRB1 and CRB3 (*p* = 0.6 and *p* = 0.13, respectively; Figure 1B). We further examined CRB2 expression across multiple cancer types (pan‐cancer) (Figure 1C) and validated its significant upregulation in ovarian cancer using GEO datasets GSE10971 and GSE66957 (*p* = 0.0061 and *p* < 0.0001, respectively; Figure 1D).

### 3.2 CRB2 Is Highly Expressed in Epithelial Ovarian Cancer and Correlates With Clinicopathological Features

**FIGURE 2:**
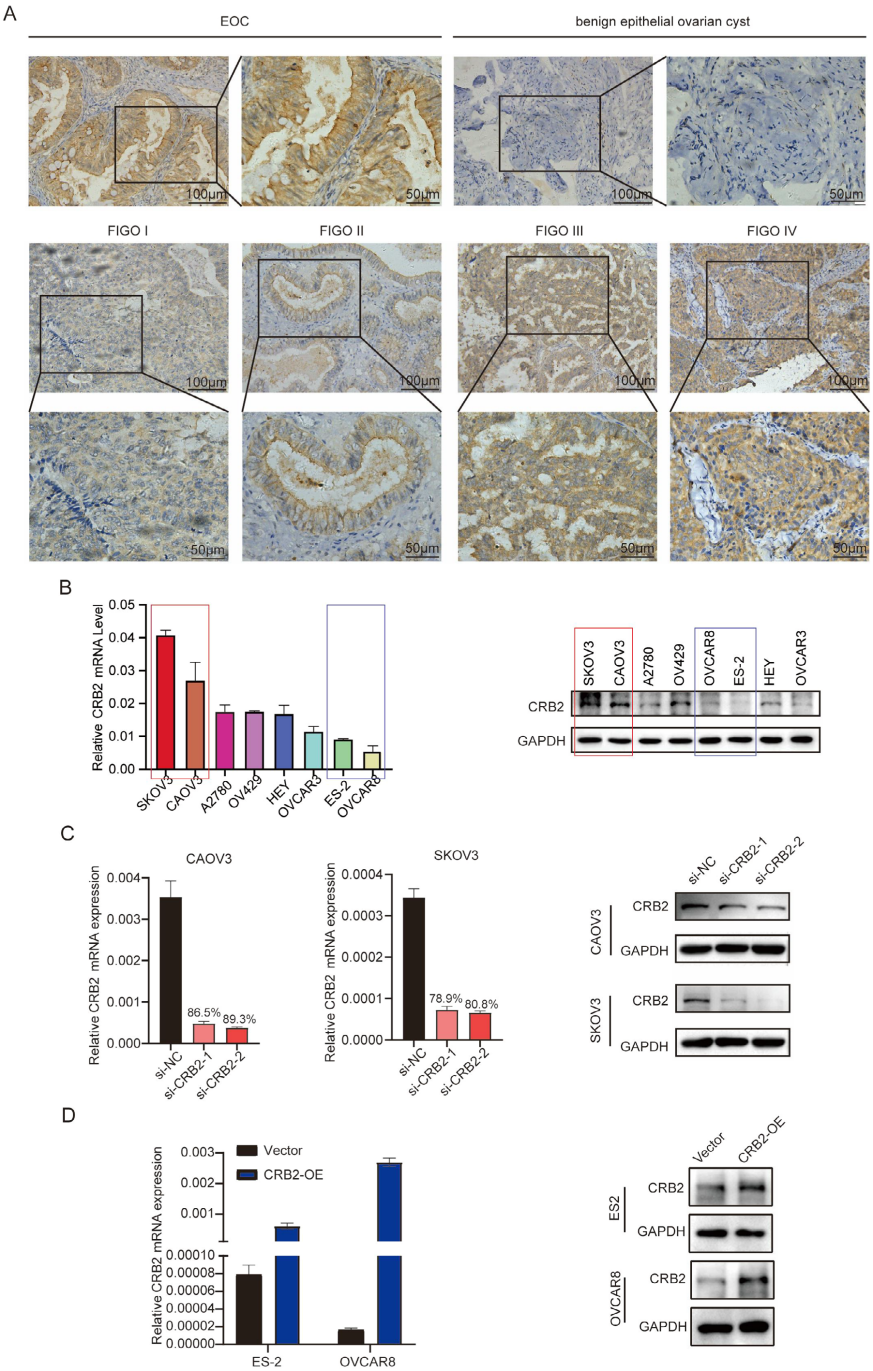
Expression of CRB2 in Ovarian Tumour Tissues and Cell Lines, and Validation of Transfection Efficiency. (A) Immunohistochemical analysis of CRB2 expression in epithelial ovarian cancer tissues at different clinical stages and in benign epithelial ovarian cyst tissues. (B) mRNA and protein levels of CRB2 in eight epithelial ovarian cancer cell lines. (C) Validation of CRB2 knockdown efficiency at both mRNA and protein levels in CAOV3 and SKOV3 cells after transient transfection with siRNA. (D) Validation of CRB2 overexpression at both mRNA and protein levels in ES2 and OVCAR8 cells after stable lentiviral transfection. Table 1. Association Between CRB2 Expression Levels and Clinicopathological Characteristics in Epithelial Ovarian Cancer [n (%)].

To investigate the clinical significance of CRB2 in ovarian cancer, immunohistochemical (IHC) analysis was performed on tissue microarrays containing samples from 154 patients with benign ovarian epithelial cysts and 174 patients with epithelial ovarian cancer (EOC). The results showed that CRB2 expression was significantly higher in ovarian cancer tissues (Figure 2A). Specifically, the proportion of high CRB2 expression was 27.3% in benign ovarian epithelial cysts, compared to 75.1% in ovarian cancer tissues (*p* < 0.001).

We further analysed the association between CRB2 expression and clinicopathological features of EOC. CRB2 expression was markedly elevated in high‐grade ovarian cancer compared to low‐grade samples; the proportion of high CRB2 expression was 55.1% in low‐grade tumours and 83.2% in high‐grade tumours (*p* < 0.001). Compared to early‐stage (I + II) patients, CRB2 expression was higher in late‐stage (III + IV) ovarian cancer, with high expression observed in 65.9% of early‐stage and 84.3% of late‐stage cases (*p* = 0.008). Additionally, CRB2 expression was significantly elevated in cases with lymph node metastasis; 69.1% of lymph node‐negative cases showed high CRB2 expression, whereas 85.9% of lymph node‐positive cases exhibited high expression (*p* = 0.017) (Table 1).

**TABLE 1:** Relationship between the expression level of CRB2 and the clinical and pathological characteristics of ovarian cancer [*n* (%)].

		CRB2 Low	CRB2 High	Total	χ2	*P* value
Type	EOC	43 (24.7)	131 (75.3)	174	75.564	< 0.001
	Ovarian cyst	112 (72.7)	42 (27.3)	154		
	Total	155	173	328		
Grade	High‐grade	21 (16.8)	104 (83.2)	125	14.937	< 0.001
	Low‐grade	22 (44.9)	27 (55.1)	49		
	Total	43	131	174		
Stage	I + II	29 (34.1)	56 (65.9)	85	7.901	0.008
	III + IV	14 (15.7)	75 (84.3)	89		
	Total	43	131	174		
lymph node	Positive	9 (14.1)	55 (85.9)	64	6.172	0.017
	Negative	34 (30.9)	76 (69.1)	110		
	Total	43	131	174		
Age	≤ 50	18 (29.0)	44 (71.0)	62	0.966	0.361
	> 50	25 (22.3)	87 (77.7)	112		
	Total	43	131	174		

Collectively, these findings demonstrate that CRB2 is significantly overexpressed in epithelial ovarian cancer tissues, and its high expression is closely associated with advanced clinical stage, higher pathological grade, and lymph node metastasis. CRB2 may serve as a potential biomarker for predicting poor prognosis in ovarian cancer patients.

### 3.3 CRB2 Promotes Proliferation, Migration, and Invasion While Inhibiting Apoptosis in Ovarian Cancer Cells

We analysed CRB2 expression at both the mRNA and protein levels in eight epithelial ovarian cancer (EOC) cell lines (Figure 2B). Among them, CAOV3 and SKOV3 cells, which exhibited relatively high CRB2 expression, were selected for CRB2 knockdown using specific siRNAs (Figure 2C). Conversely, ES2 and OVCAR8 cells, which had relatively low CRB2 expression, were used to generate stable CRB2‐overexpressing cell lines via lentiviral transduction (Figure 2D). After confirming the efficiency of CRB2 knockdown and overexpression, we conducted a series of functional assays.

**FIGURE 3:**
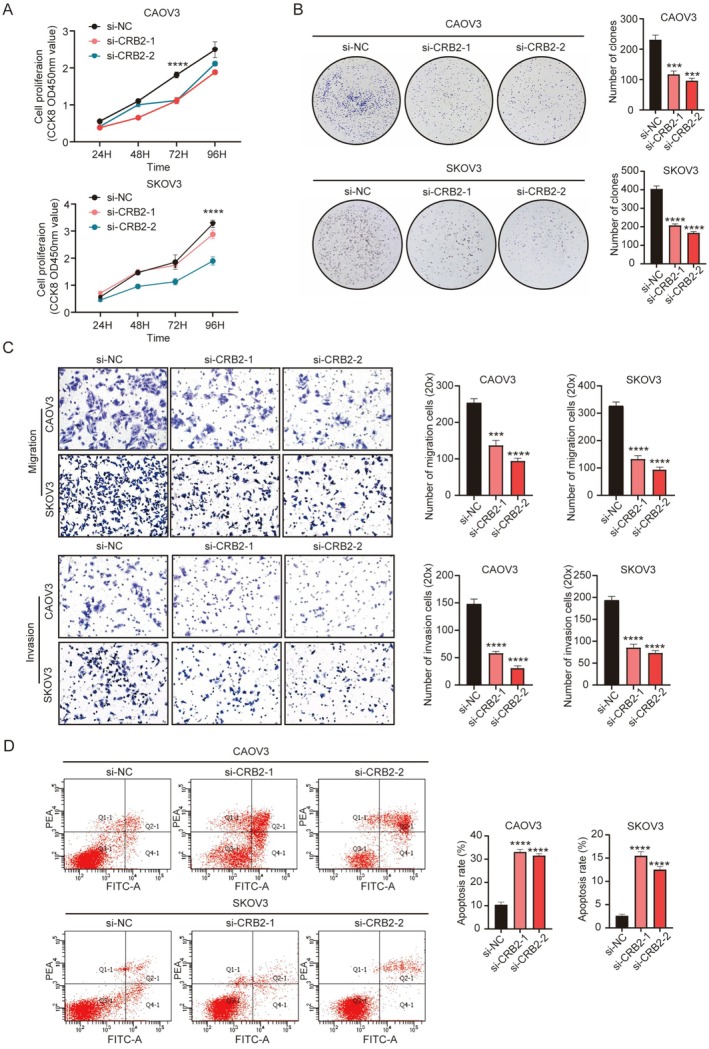
Biological functions of CRB2 in epithelial ovarian cancer cells (siRNA knockdown group). (A, B) CCK‐8 and colony formation assay showed that knockdown of CRB2 significantly inhibited ovarian cancer cell proliferation. (C) Transwell migration and invasion assays revealed that CRB2 knockdown markedly suppressed the migratory and invasive capabilities of ovarian cancer cells. (D) Flow cytometry apoptosis assay demonstrated that CRB2 knockdown significantly promoted apoptosis in ovarian cancer cells. (*P < 0.05, **P < 0.01, ***P < 0.001, ****P < 0.0001).

First, CRB2 was knocked down in CAOV3 and SKOV3 cells, followed by assessment of cell proliferation, migration, invasion, and apoptosis. Knockdown of CRB2 significantly inhibited the proliferative capacity of ovarian cancer cells: In the CCK‐8 assay, both knockdown cell lines showed markedly reduced proliferation at 72 and 96 h post‐treatment (*p* < 0.0001, Figure 3A). Colony formation assays also revealed a significant decrease in colony numbers compared to controls (P_(CAOV3)_ < 0.001, P_(SKOV3)_ < 0.0001, Figure 3B). In Transwell migration and invasion assays, quantitative analysis of cells that migrated or invaded through the membrane indicated that CRB2 knockdown significantly suppressed both migration and invasion abilities (P_(CAOV3‐Migration)_ < 0.001, P_(CAOV3‐Invasion)_ < 0.0001, P_(SKOV3‐Migration)_ < 0.0001, P_(SKOV3‐Invasion)_ < 0.0001, Figure 3C). Flow cytometry‐based apoptosis assays demonstrated that silencing CRB2 expression markedly increased the apoptosis rate in ovarian cancer cells (*p* < 0.0001, Figure 3D).

**FIGURE 4:**
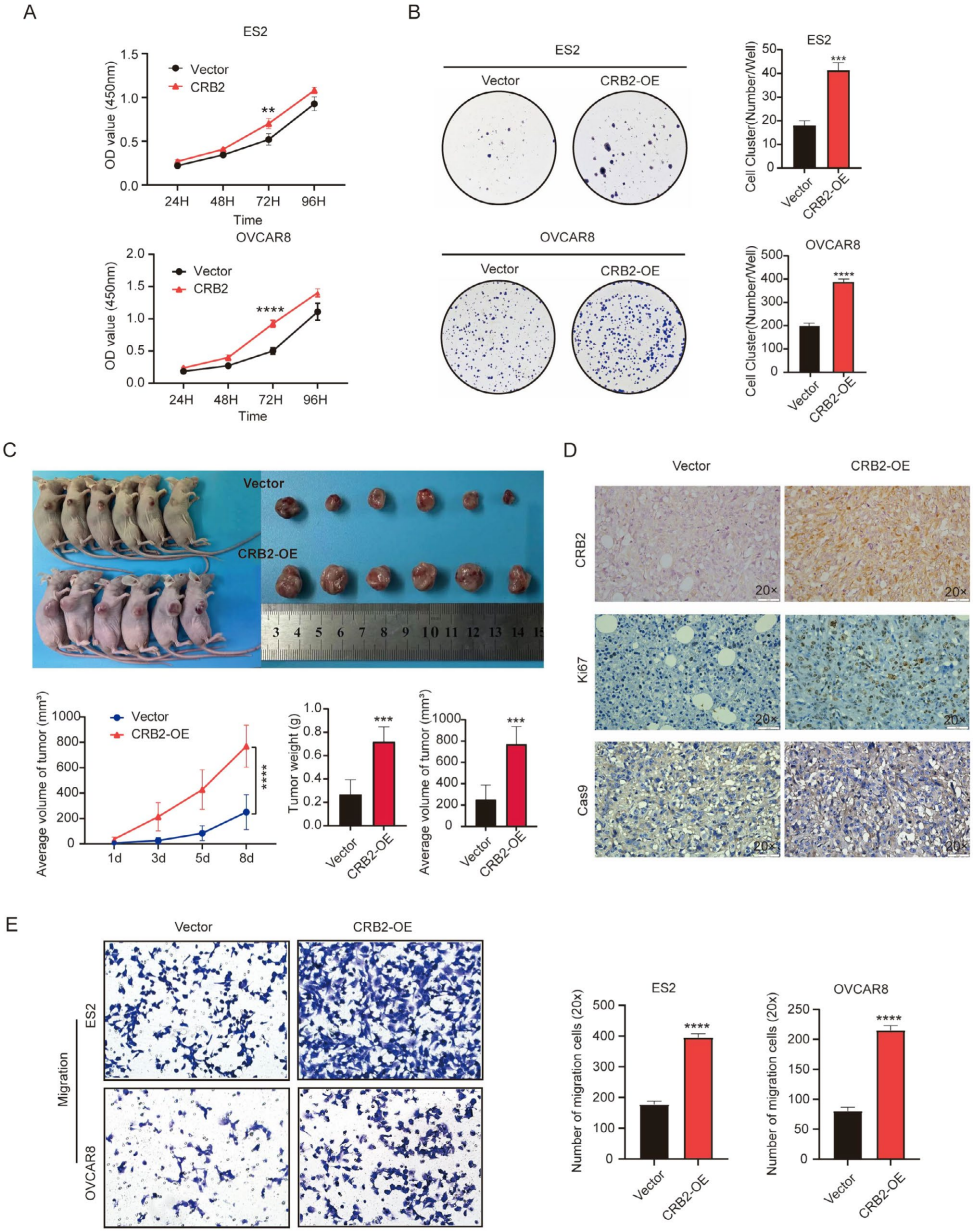
Biological functions of CRB2 in epithelial ovarian cancer cells (overexpression group). (A, B) CCK‐8 and colony formation assays showed that CRB2 overexpression significantly promoted ovarian cancer cell proliferation. (C) Subcutaneous xenograft experiments in nude mice demonstrated that CRB2 overexpression markedly enhanced in vivo tumour growth. (D) Immunohistochemical analysis of xenograft tumours revealed that CRB2 overexpression significantly increased cell proliferation (Ki‐67) and decreased apoptosis (Caspase‐9) in vivo. (E) Transwell migration and invasion assays indicated that CRB2 overexpression significantly enhanced the migratory and invasive capabilities of ovarian cancer cells. (*P < 0.05, **P < 0.01, ***P < 0.001, ****P < 0.0001).

We further validated the biological functions of CRB2 using stable CRB2‐overexpressing ovarian cancer cell lines. The results demonstrated that overexpression of CRB2 significantly enhanced the proliferative capacity of ovarian cancer cells. In the CCK‐8 assay, both CRB2‐overexpressing cell lines showed markedly increased proliferation at 72 h (P_(ES2)_ < 0.01, P_(OVCAR8)_ < 0.0001, Figure 4A). Similarly, colony formation assays showed that CRB2‐overexpressing cells formed significantly more colonies than the control group (P_(ES2)_ < 0.001, P_(OVCAR8)_ < 0.0001, Figure 4B). To evaluate tumorigenicity in vivo, subcutaneous xenograft experiments were performed using the ES2 CRB2‐overexpressing stable cell line. The results showed that tumours derived from CRB2‐overexpressing cells grew significantly faster than those in the control group (*p* < 0.01, Figure 4C). The final tumour weight and volume were both significantly increased in the CRB2‐overexpressing group compared to the control group (*p* < 0.001, Figure 4C). Immunohistochemical staining of tumour sections confirmed higher CRB2 expression in the overexpression group, along with a marked increase in the proliferation marker Ki‐67 and a notable decrease in the apoptosis marker Caspase‐9 (Figure 4D). Furthermore, Transwell migration and invasion assays showed that CRB2‐overexpressing ovarian cancer cells exhibited significantly enhanced migration and invasion abilities compared to controls (P_(ES2‐Migration)_ < 0.0001, P_(ES2‐Invasion)_ < 0.001, P_(OVCAR8‐Migration)_ < 0.0001, P_(OVCAR8‐Invasion)_ < 0.001, Figure 4E).

The above cellular functional experiments indicate that CRB2 promotes the proliferation, migration, and invasion of ovarian cancer cells while inhibiting their apoptosis.

### 3.4 CRB2 Affects Cell Polarity and Promotes Directional Migration in Ovarian Cancer Cells

**FIGURE 5:**
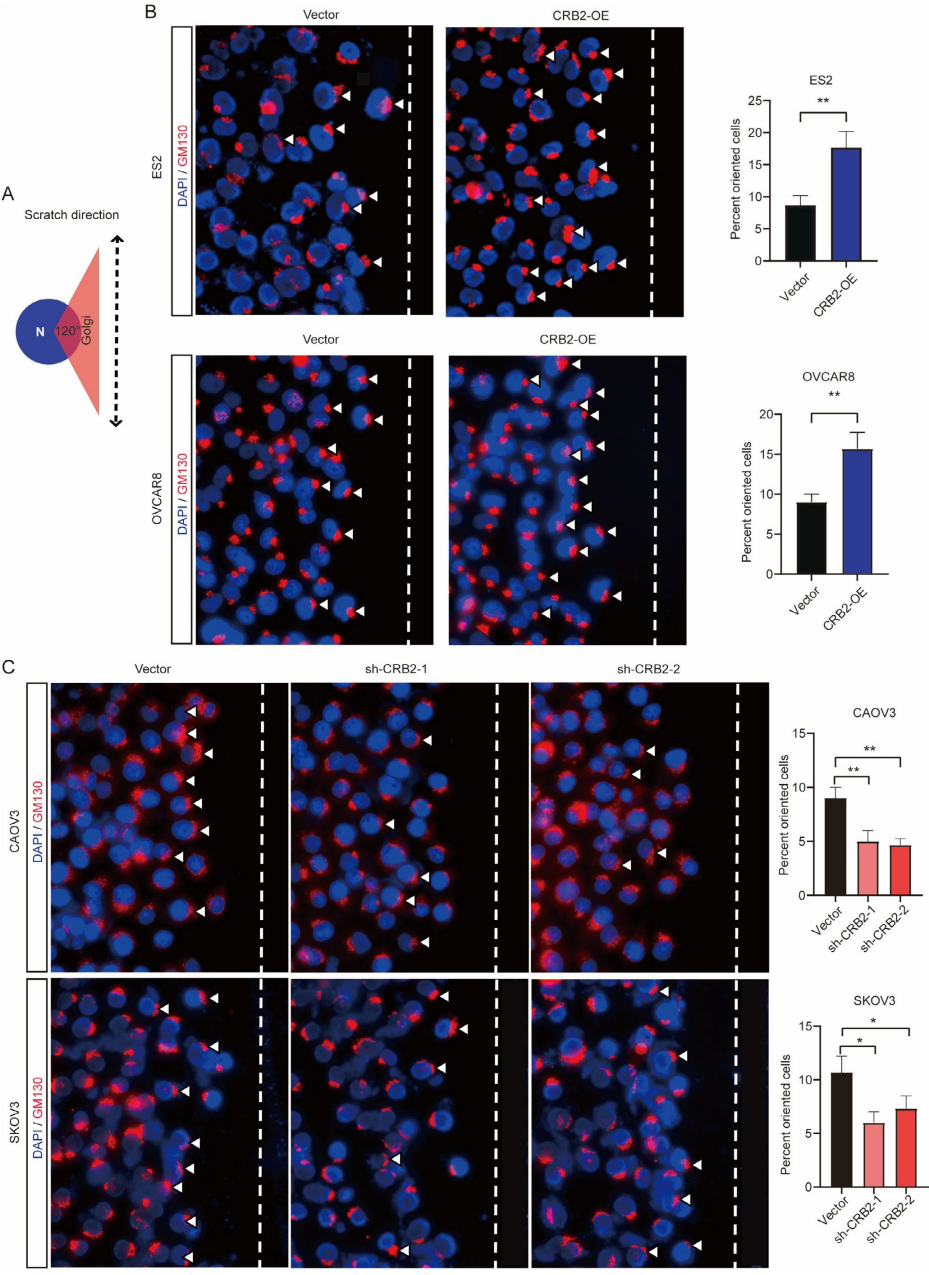
CRB2 regulates cell polarity and promotes directional migration in ovarian cancer cells. (A) Schematic diagram illustrating the measurement of directional migration rate. (B) Immunofluorescence co‐localisation analysis showing changes in cell polarity and directional migration rate after CRB2 knockdown in ovarian cancer cells. (C) Immunofluorescence co‐localisation analysis showing changes in cell polarity and directional migration rate following CRB2 overexpression. (*P < 0.05, **P < 0.01, ***P < 0.001, ****P < 0.0001).

To further investigate whether CRB2 influences polarity changes in ovarian cancer cells, planar scratch assays followed by immunofluorescence staining were performed. Golgi apparatus localisation, marked by GM130, was used to assess cell polarity. Golgi positioning within a 120° sector in front of the nucleus was defined as indicative of directional migration. As shown in Figure 5A, CRB2 overexpression significantly increased the percentage of cells exhibiting directional migration compared to the control group (*p* < 0.01, Figure 5B). Conversely, CRB2 knockdown markedly reduced the directional migration rate (*p* < 0.01, Figure 5C). These findings suggest that CRB2 regulates ovarian cancer cell polarity and promotes directional migration.

### 3.5 CRB2 Promotes Cancer Progression by Activating the Wnt/PCP Pathway

**FIGURE 6:**
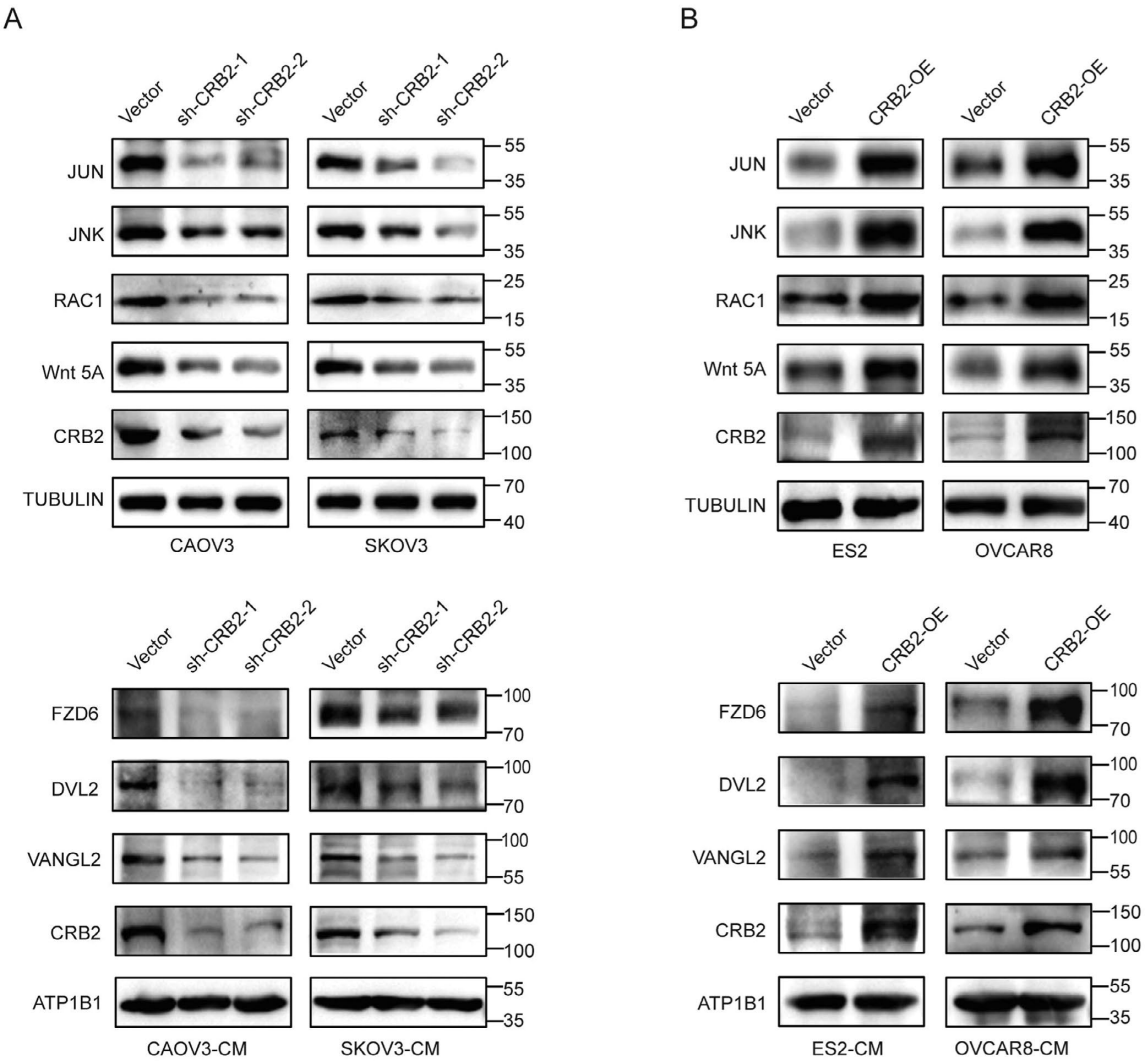
Preliminary investigation of the molecular mechanism by which CRB2 promotes ovarian cancer progression. (A) Expression analysis of Wnt/PCP pathway‐related proteins in ovarian cancer cells following CRB2 knockdown. (B) Expression analysis of Wnt/PCP pathway‐related proteins in ovarian cancer cells following CRB2 overexpression.

Planar scratch‐based immunofluorescence assays demonstrated that CRB2 regulates polarity changes in ovarian cancer cells. To further explore the underlying mechanism, we examined key proteins associated with the Wnt/planar cell polarity (Wnt/PCP) signalling pathway. Upon CRB2 knockdown, the expression levels of Wnt5a, RAC1, JNK, and JUN were markedly reduced. Additionally, after isolating membrane and cytoplasmic protein fractions, we observed decreased membrane‐associated levels of FZD6 and VANGL2, and a significant reduction in DVL2 expression (Figure 6A). Conversely, CRB2 overexpression resulted in increased expression of Wnt5a, RAC1, JNK, and JUN, along with elevated membrane localisation of FZD6 and VANGL2 and significantly increased DVL2 levels (Figure 6B).

These findings suggest that CRB2 likely initiates Wnt5a‐mediated activation of the membrane receptor FZD6, thereby promoting the recruitment of VANGL2 and DVL2, and subsequently triggering a RAC1‐JNK‐JUN signalling cascade. Collectively, these results indicate that CRB2 facilitates epithelial ovarian cancer progression by promoting cell polarity changes through activation of the Wnt/PCP signalling pathway.

## 4 Discussion

Cell polarity refers to the asymmetric organisation of cellular components, including proteins, lipids, and organelles, which is essential for maintaining the structural and functional integrity of epithelial tissues [25, 26]. Proper cell polarity ensures controlled cell proliferation, differentiation, and tissue architecture, whereas loss or disruption of polarity is a hallmark of epithelial‐to‐mesenchymal transition (EMT) and contributes to tumour initiation, progression, and metastasis [25, 27, 28]. Dysregulation of polarity proteins can drive uncontrolled proliferation, enhanced invasion, and aberrant migration—key characteristics of malignant progression [27, 29]. Members of the Crumbs family play pivotal roles in establishing apical–basal polarity in epithelial cells [26, 30]. Altered CRB expression can compromise polarity, thereby facilitating directional migration and invasive behaviour of ovarian cancer cells [26, 28, 30].

Comprehensive transcriptomic analyses of the TCGA and GTEx datasets revealed that CRB2 and CRB3 were markedly upregulated in ovarian cancer tissues compared with normal ovarian samples, whereas CRB1 showed no significant difference. Kaplan–Meier Plotter–based survival evaluation further demonstrated that only elevated CRB2 expression correlated with unfavourable prognosis in patients with ovarian cancer. Accordingly, CRB2 was selected for subsequent functional characterisation in this study.

Crumbs Cell Polarity Complex Component 2 (CRB2) is a type I transmembrane protein associated with cell polarity regulation [31]. It functions as a novel regulator of embryonic stem cell‐derived neural progenitor cells. During neural differentiation, CRB2 overexpression upregulates the expression of other apical polarity proteins and promotes cell proliferation, and it is essential for the stabilisation of these polarity proteins [32]. Mutations in the CRB2 gene have been reported to cause focal segmental glomerulosclerosis [31, 33], ventriculomegaly, and cystic kidney disease [34, 35]. Additionally, CRB2 can trigger epithelial–mesenchymal transition (EMT) in retinal pigment epithelial (RPE) cells, contributing to retinitis pigmentosa [36]. Moreover, CRB2 contributes to glioblastoma malignancy by activating the NF‐κB signalling cascade [37]. Loss or disruption of polarity is a hallmark of EMT and contributes to tumour progression, as previously reported [28]. However, its potential role and clinical relevance in ovarian cancer have not yet been defined.

Tumour differentiation grade and pathological stage are independent prognostic factors for patients with ovarian cancer, with advanced‐stage disease associated with poorer outcomes and a lower 5‐year survival rate [38, 39]. In this study, tissue microarray‐based immunohistochemistry was used to assess CRB2 expression in benign epithelial ovarian cysts and epithelial ovarian cancer tissues. Our results demonstrated that CRB2 was significantly overexpressed in epithelial ovarian cancer tissues, and its high expression was closely associated with advanced clinical stage, high pathological grade, and lymph node metastasis. These findings suggest that CRB2 may serve as an effective prognostic biomarker for ovarian cancer.

To further investigate the biological role of CRB2 in ovarian cancer, we performed a series of in vitro and in vivo functional experiments by silencing or overexpressing CRB2 in ovarian cancer cells. The results revealed that CRB2 significantly promoted cell proliferation, migration, and invasion, while inhibiting apoptosis. The Golgi apparatus, a critical organelle in eukaryotic cells responsible for protein modification, sorting, and transport, undergoes dynamic morphological changes that are essential for its function, cell cycle regulation, and the establishment of cell polarity [40]. We employed immunofluorescence staining following scratch assays to analyse changes in cell polarity based on the subcellular localisation of the Golgi apparatus. The results showed that CRB2 overexpression significantly increased the directional migration of ovarian cancer cells, whereas CRB2 silencing significantly reduced it, indicating that CRB2 regulates cell polarity and promotes directional migration.

The Wnt/Planar Cell Polarity (PCP) pathway is a non‐canonical Wnt signalling cascade that plays a central role in regulating cell polarity and migration [41, 42]. In Drosophila, PCP is established through the asymmetric localisation of two protein complexes: The Frizzled–Dishevelled–Diego–Flamingo complex and the Van Gogh (Strabismus)–Prickle complex [43]. In human cancers, aberrant PCP signalling contributes to disrupted polarity and enhanced invasiveness, consistent with prior observations of EMT‐related signalling [28, 43]. Ligands such as Wnt5a, Wnt7a, and Wnt11 activate the pathway by binding to atypical Frizzled receptors (e.g., FZD3/FZD6) and co‐receptors, including protein tyrosine kinase 7 (PTK7), receptor tyrosine kinases ROR1/2, and RYK [44]. Core PCP components include VANGL1, VANGL2, CELSR1–3, DVL1–3, PRICKLE1–2, and ANKRD6 (homologues of Diego) [43].

Previous studies have shown that Wnt5a is upregulated in various human cancers, including colorectal [45], gastric [46], lung [47], and ovarian cancers [48]. In ovarian cancer, Wnt5a enhances the adhesion and invasive migration of tumour cells to peritoneal mesothelial cells, promoting peritoneal implantation [49]. In gastric cancer, Wnt5a signalling is mediated by FZD3/FZD6 receptors and the ROR2 co‐receptor. VANGL2 functions as a scaffold protein at the cell membrane and is essential for the assembly of PCP complexes [50]. Upon activation, the Wnt/PCP pathway promotes downstream signalling via DVL proteins, leading to the activation of RHO GTPases and subsequently RHO‐associated kinase (ROCK), as well as RAC GTPase‐mediated activation of c‐Jun N‐terminal kinase (JNK), which enhances transcription of AP‐1–dependent genes. These cascades regulate cytoskeletal remodelling and cell motility [51].

To elucidate the molecular mechanism by which CRB2 promotes ovarian cancer progression, we examined the expression of key proteins in the Wnt/PCP pathway. Our results showed that CRB2 upregulated Wnt5a, leading to the activation of the membrane receptor FZD6, recruitment of VANGL2 and DVL2, and subsequent activation of the RAC1–JNK–JUN cascade. These findings suggest that CRB2 may regulate ovarian cancer cell polarity and directional migration through activation of the Wnt/PCP pathway.

Taken together, our results identify CRB2 as a key promoter of epithelial ovarian cancer progression through modulation of polarity‐associated Wnt/PCP signalling, highlighting its potential as a prognostic marker and therapeutic target.

## Author Contributions

**Chunlin Tao:** conceptualization, software, resources, methodology, data curation, validation, writing – original draft, funding acquisition. **Shaojing Li:** conceptualization, methodology. **Duohe Sun:** resources, formal analysis. **Jun Zhou:** investigation, validation. **Yuanping Chen:** methodology, formal analysis. **Rong Zhang:** conceptualization, methodology, validation, data curation, project administration, supervision, funding acquisition. **Xiaoge Ni:** methodology, formal analysis, investigation, validation, data curation, project administration, writing – review and editing, supervision.

## Funding

This study was supported by the National Natural Science Foundation of China (No. 82472923) and the Social Science and Technology Development Fund of Fengxian District Science and Technology Commission, Shanghai (No. 20241316).

## Ethics Statement

The collection of patient tissue specimens and the animal study protocol were approved by the Ethics Committee of the South Campus of Shanghai Sixth People's Hospital Affiliated to Shanghai Jiao Tong University (Approval No. 2025‐KY‐59‐02). All clinical samples used in this study were obtained with informed consent from the patients.

## Consent

Written informed consent for publication was obtained from all participants.

## Conflicts of Interest

The authors declare no conflicts of interest.

## Data Availability

Research data are not shared.
